# An historical overview over Pharmacovigilance

**DOI:** 10.1007/s11096-018-0657-1

**Published:** 2018-06-15

**Authors:** Giulia Fornasier, Sara Francescon, Roberto Leone, Paolo Baldo

**Affiliations:** 1 Pharmacy Unit, Centro di Riferimento Oncologico CRO Aviano, National Cancer Institute - IRCCS, Aviano, Italy; 2 Pharmacology Unit, Department of Diagnostics and Public Health, University of Verona, Verona, Italy

**Keywords:** Adverse drug reactions, History, Legislation, Pharmacovigilances, Signal detection, Thalidomide

## Abstract

Pharmacovigilance started about 170 years ago, although it was not yet named as such at that time. It is structured activity in the professional health field, with important social and commercial implications aimed at monitoring the risk/benefit ratio of drugs, improving patient’s safety and the quality of life. In this commentary we report the milestones of pharmacovigilance up to the present day, in order to understand all the steps that have characterized the historical evolution; from the first reports, which were essentially letters or warnings sent by clinicians to publishers of important and famous scientific journals, up to today’s modern and ultra-structured electronic registries. The historical phases also help us to understand why pharmacovigilance helped us to achieve such important results for man’s health and for pharmacology itself, and to identify the challenges that await Pharmacovigilance in future years.

Pharmacovigilance (PV) is defined by the European Commission (EU) as the “Process and science of monitoring the safety of medicines and taking action to reduce the risks and increase the benefits of medicines”. The international PV systems aim to monitor the risk/benefit ratio of drugs as well as improve patients’ safety and their quality of life. PV activities include: collecting and managing data on the safety of medicines, looking at individual case reports to detect new “signals”, *pro*-*active* risk management to minimize any potential risk associated with the use of medicines, communicating and informing stakeholders and patients. This seamless post-marketing surveillance, which is primarily aimed at protecting the public, allows CAs (Controlling Authorities) to modify—on the basis of newly discovered signals—the Summary Product Characteristics (SPC), released by the Marketing Authorization Holder (MAH) for any new medicinal product at the first boot into the market [1].

The etymological roots for the word “pharmacovigilance” are: *Pharmakon* (Greek) = medicinal substance, and *Vigilia* (Latin) = to keep watch.

In this short article, we describe the milestones (as represented in Fig. 1) that led to the evolution of Pharmacovigilance activities in the last century.

**Fig. 1:**
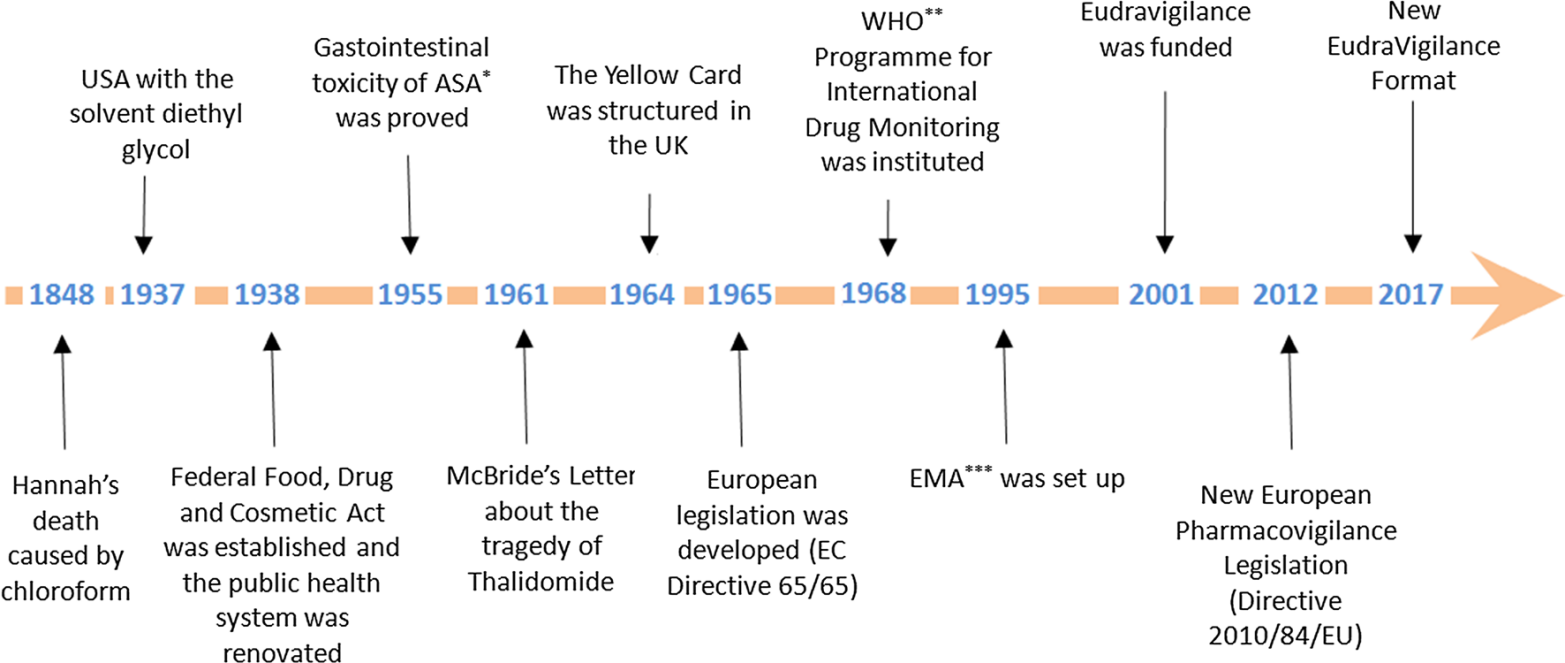
Timeline of the historical evolution of Pharmacovigilance. *ASA: acetylsalicylic acid; **WHO: World Health Orgnaisation; ***EMA: European Medicines Agency

We intentionally excluded a part of scandals (e.g. inhibitors of cyclooxygenase types 2 because of cardiovascular adverse reactions), because they were mainly due to incorrect marketing or inappropriate information campaigns by pharmaceutical companies [2].

The history of Pharmacovigilance started 169 years ago, on Jan 29, 1848, when a young girl (Hannah Greener) from the north of England died after receiving chloroform anesthetic before removal of an infected toenail. Sir James Simpson had discovered that chloroform was a safer and powerful anesthetic, and he had introduced it in clinical practice. The causes of Hannah’s death was investigated to understand what happened to Hannah, but it was impossible to identify what killed her. Probably she died of a lethal arrhythmia or pulmonary aspiration [3].

As a result of other deaths and alerts raised by the clinicians and the public about the safety of anesthesia, *The Lancet* Journal established a commission to take on this problem. The commission exhorted English doctors, including the doctor in colonies, to report deaths caused by the anesthesia. The results were published in The Lancet in 1893 [4].

The US Federal Food and Drug Act was formed on June 30, 1906, and it established that drugs must be pure and free of any contamination. Furthermore, in 1911, this organization forbade false therapeutic indications of drugs [4]. In 1937, there were 107 deaths in the USA, because of the use of sulfanilamide elixir, containing diethyl glycol as the solvent. This solvent was considered the cause of deaths, but the manufactory companies were not aware about its toxicity at that time [3, 5, 6]. Consequently, the Federal Food, Drug and Cosmetic Act was established in 1938; its aim was to renovate the public health system. Indeed, the new system foresaw that the safety of drugs should be demonstrated before their market approval, and introduced the possibility of conducting factory inspections [7]. In 1938, Douthwaite supposed that acetylsalicylic acid (ASA) could cause melena [8]. The study of the gastrointestinal toxicity of ASA showed different outcomes. However, in 1955, it was proved that ASA can cause gastrointestinal diseases and therefore it is currently contraindicated in patients with gastrointestinal ulcers [9].

In 1961, a big change of European Pharmacovigilance happened following the tragedy of Thalidomide. Dr. McBride, an Australian doctor, wrote a letter to the editor of the Lancet Journal, in which he suggested a connection between congenital malformation of babies and thalidomide. In fact, he observed that the incidence of congenital malformations of babies (1.5%) had increased up to 20% in women who had taken thalidomide during pregnancy [10]. At the same time, during a Pediatric Convention in Germany Dr. Lenz suggested a correlation between malformations and thalidomide and his suspect was published in a German Journal (Welt am Sonnatag) [11]. In 1973, a retrospective study showed the correlation between the congenital malformations of babies and the ingestion of thalidomide during pregnancy [12]. In USA, the tragedy of thalidomide was not observed, because Dr. Kelsey showed strong doubts about the safety of thalidomide during pregnancy [5]. The tragedy of thalidomide brought to light many problems and critical issues, in particular, the reliability of animal tests, the behavior of the industrial company, and the importance of monitoring the drugs after their marketing. In particular, this tragedy changes the system of Pharmacovigilance, because the spontaneous reporting of adverse drug reactions became systematic, organized, and regulated. This letter already contained all of the elements needed to generate a spontaneous reporting and to establish a cause-effect relationship between the adverse event and the drug (Fig. 2) [13]. In 1964, the “Yellow card” (YC) was structured in the UK. YC is a specific form to compile a spontaneous report of drug toxicity [14]. In USA (1962), the amendment, requiring safety and efficacy data of drugs before premarketing submission, was approved. As a result of this amendment, the safety data have to include also teratogenicity test in three different animals [5]. In Europe (1965), the disaster of thalidomide stimulated the development of a European legislation with the EC Directive 65/65 [15]. In 1966, a pilot study of Boston Collaborative Drug Surveillance Program started. It was the first group to conduct epidemiologic researches to quantify the potential adverse effects of drugs utilizing in-hospital monitoring and had an essential role in the development and application of methods in drug epidemiology [16]. In 1968, the WHO Programme for International Drug Monitoring was instituted and ten members participated in this program (Australia, UK, USA, Germany, Canada, Ireland, Sweden, Denmark, New Zealand, and Netherlands). Italy participated in this program in 1975 [17]. Many studies of observed adverse drug reactions were conducted between 1968 and 1982 [3]. In 1992, the European Society of Pharmacovigilance (ESoP) was funded, turned into the International Society of Pharmacovigilance (IsoP). The aims of this society were to promote Pharmacovigilance, and enhance all aspects of the safe and proper use of medicines [18]. In 1995, the European Medicines Agency (EMA) was set up [19]. In 2001, EudraVigilance was funded. It is the official European database for managing and analyzing information on suspected adverse reactions to medicines which have been authorized for the market or being studied in European clinical trials [20]. A major change in European Pharmacovigilance was observed with the new legislation (Directive 2010/84/EU), in 2012 [20]. The main changes in the new legislation were [21]:

- Modification of the definition of adverse drug reactions (ADR);
- Greater involvement of patients and citizens in Pharmacovigilance activities;
- Strengthening of the Eudravigilance database containing reports of suspected reactions reported by all EU Member States;
- Increasing transparency and timeliness of important information on Pharmacovigilance problems;
- Obligation of “additional monitoring” for the products contained in the specific list kept by the EMA;
- Possibility to impose further safety and/or efficacy studies on the certificates of marketing authorization at the time of granting the trust;
- Establishment within the EMA of the Pharmacovigilance Risk Assessment Committee (PRAC).

**Fig. 2:**
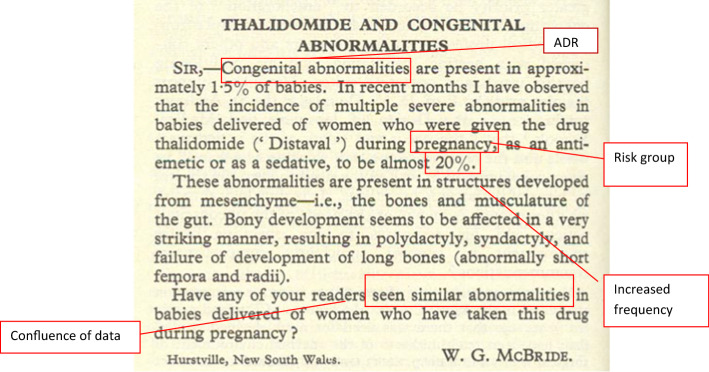
McBride’s letter and important elements for generating spontaneous reporting

In particular, the most relevant change consists in the new definition of ADR: “A response to a medicinal product which is noxious and unintended”. In fact, with this definition were covering any adverse event following the use of a medicine, also medication errors and uses outside the terms of the marketing authorization, including the misuse and abuse of the medicinal product.

Furthermore, the new legislation set-up measures to facilitate the performance of PV, called the Good Pharmacovigilance Practices (GVP). The guideline on GVP is divided into two categories: modules covering major Pharmacovigilance processes and product- or population-specific considerations. This last category is available for vaccines and biological medicinal products. In this guideline there are also special chapters dedicated to special areas, namely pregnancy and breast-feeding (P III) and geriatric population (P V) [22].

In November 2017, the new EudraVigilance format was launched; in particular, the marketing authorizations will have extended access to the EudraVigilance database to support the fulfillment of their Pharmacovigilance obligations. These obligations include the continuous monitoring of EudraVigilance data and the communication of validated signals to the Agency and national regulatory authorities, as outlined in Commission Implementing Regulation (EU) N. 520/20121 [19].
